# Structural heat adaptation and education in urban settings (SHAPES trial): study protocol for a cluster randomised controlled trial

**DOI:** 10.1136/bmjopen-2026-120115

**Published:** 2026-08-11

**Authors:** Jai K Das, Syeda Kanza Naqvi, Ana Bonell, Christopher Burman, Joseph Augustin, Shahmeer Qamar, Arjumand Rizvi, Imran Ahmed Chauhdary, Anjum Abbas Naqvi, Muhammad Khan Jamali, Hassan Naqvi, Farhana Tabassum, Simon Cousens, Zulfiqar A Bhutta

**Affiliations:** 1 Institute for Global Health and Development, The Aga Khan University, Karachi, Pakistan; 2 Department of Paediatrics and Child Health, The Aga Khan University, Karachi, Pakistan; 3 Medical Research Council Unit The Gambia at the London School of Hygiene & Tropical Medicine, Banjul, Gambia; 4 London School of Hygiene & Tropical Medicine, London, UK; 5 Bartlett School of Architecture, University College London, London, UK; 6 Centre of Excellence in Women and Child Health, The Aga Khan University, Karachi, Pakistan; 7 Centre for Global Child Health, Hospital for Sick Children, Toronto, Ontario, Canada

**Keywords:** Climate Change, Community-Based Participatory Research, Primary Prevention, PUBLIC HEALTH, Quality of Life

## Abstract

**Background:**

Extreme heat events are intensifying worldwide due to climate change, posing significant health risks and economic challenges. The urban heat island effect poses extra challenges to the inhabitants of cities, especially in the global South.

**Methods:**

This trial aims to evaluate the effectiveness of a comprehensive resilience and heat adaptation bundle (ReHAB) that integrates behavioural and structural interventions to reduce heat-related morbidity and mortality and improve internal ambient conditions in urban settings of Pakistan. This would be a cluster randomised controlled trial where 22 clusters comprising approximately 70–80 households per cluster will be randomly assigned to intervention and control arms in a 1:1 ratio. The intervention ReHAB is a combination of community education and awareness and targeted structural modifications, including reflective paints, shading, sustained energy/power, improved ventilation, and community shaded spaces. Data on health and behaviours will be collected via wearable devices and household surveys and environmental sensors will be used to capture indoor and outdoor thermal conditions.

**Expected outcomes:**

The findings from this trial will provide evidence for the effectiveness of the ReHAB bundle on indoor ambient environments and health and help policy recommendations for urban areas, especially for the global south.

**Ethics and dissemination:**

The study has received ethical approval from the Aga Khan University (AKU) (Ref: 2025-11548-35626) and the Pakistan National Bioethics Committee (Ref: 4–87/NBCR-1065/23/1736). Written informed consent will be obtained from all participants before enrolment. Referral pathways to healthcare facilities will be established to ensure timely management of complications. Findings will be disseminated through peer-reviewed publications, scientific conferences and engagement with policymakers and public health stakeholders. Results will also be shared with participants and communities through meetings and informal sessions to raise awareness and support evidence-based heat adaptation.

**Trial registration number:**

NCT06991543.

**STRENGTHS AND LIMITATIONS OF THIS STUDY**

- Use of weather stations, environmental devices, personal wearable devices and other data collection measures will enable triangulation of household environment, individual exposures, health outcomes and physiological profiling.
- Blinding of participants and field teams is not feasible because of the nature of the structural and behavioural interventions.
- We anticipate challenges in compliance with wearable devices and device discomfort during hot weather.

## Background

 Anthropogenic climate change, defined as long-term shifts in temperatures and weather patterns, has significantly raised global average temperatures and extreme weather events, particularly heatwaves, have increased in frequency and intensity in regions of South Asia, West Africa, the Middle East and North Africa.^1 2^ Approximately 40% of the global population resides in regions where annual temperatures exceed 30 °C^3^ and deadly heatwaves in India and Pakistan are approximately 30 times more likely due to climate change.^4^

Extreme heat enhances the risk of morbidity and mortality, particularly among the most vulnerable,^5–10^ and between the years 2000 and 2019, heat-related deaths have averaged 489 000 annually, with Asia and Europe accounting for 45% and 36% of these fatalities, respectively.^4 11^ Beyond its direct health effects, extreme heat undermines daily work and the quality of life by limiting outdoor work and social activities and exacerbating stress which poses serious risks to mental health and psychosocial well-being.^12 13^

In the major cities of Pakistan, rapid urbanisation and expansion of informal settlements have led to the rise in dense concrete construction and loss of green spaces, with compounding strain on water and energy supplies further exacerbating heat stress,^14^ known as the urban heat island (UHI) effect.^15–19^ Satellite-derived land surface temperature data from 2001 to 2022 shows that the densely built-up areas of the cities of Lahore, Karachi and Faisalabad in Pakistan can experience elevated temperature differences of up to 4 °C during the day, with even greater differences at night when compared with its peripheral.^20^ In addition, low-income urban settlements are not homogeneous and often comprise a mix of housing typologies, including reinforced concrete (RCC) structures, semi-permanent dwellings and in some cases, mud-brick housing. Variations in building materials, ventilation and housing density can substantially influence indoor heat exposure and vulnerability to extreme heat. There has also been a major architectural shift, where modern concrete structures have replaced traditional designs. Colonial-era houses were oriented to face the sea breeze, featured high ceilings, deep verandas (‘baraamdahs’) and aligned window openings that encouraged through-drafts.^21–23^

A scoping review found that most adaptation research comes from the Global North, limiting evidence from Low and Middle Income Countries (LMICs).^24^ In June 2015, Karachi experienced a catastrophic heatwave that resulted in over 1200 deaths in a day, disproportionately impacting low-income populations, thus exposing vulnerabilities of the urban infrastructure and health systems.^25^ This led to the formulation of the ‘Karachi Heatwave Management Plan’ in 2017 which comprised early warning systems and emergency coordination mechanisms.^26^ Despite these advances, the long-term community-level resilience remains neglected, a randomised controlled trial by Razzak *et al* demonstrated that community-based education can significantly improve heat literacy and modestly reduce heat-related health events in low-income areas^27^ and similarly, the Ahmedabad Heat Action Plan in India has shown a reduction in heat-related mortality through early warnings, public awareness campaigns and cooling centres.^28^

Most strategies remain fragmented with a focus on early warnings or public messaging and rarely focus on integrating structural solutions that can address the physical determinants of heat exposure, especially in informal urban settlements. There is emerging evidence on the effectiveness of improved vegetation, reflective paints, cool pavements, heat education and early warning systems which have been tested in limited settings.^27^ The Structural Heat Adaptation and Education in Urban Settings trial is therefore timely and essential and represents one of the first rigorously designed, cluster-randomised evaluations of a comprehensive community-based heat adaptation package that integrates behavioural, structural and community-led components.

## Methodology

### Objective

#### Primary

1. To assess the feasibility and effectiveness of a resilience and heat adaptation bundle (ReHAB) on heat-related health risks for vulnerable populations in urban settings of Pakistan.
2. To assess the effectiveness of ReHAB on the quality of life of vulnerable populations in urban settings of Pakistan.

#### Secondary

1. To evaluate the effectiveness of ReHAB on indoor heat in urban settings.
2. To evaluate the impact of ReHAB on key physiological variables (sleep quality, heart rate, physical activity and energy expenditure) in adults residing in urban settings.

### Study setting

Karachi has a tropical semi-arid climate where summers are sweltering, humid and evenings are breezy, while winters are brief, pleasant and arid, with clear skies most of the year and daily average temperatures ranging from 13°C to 34°C.^28^ In recent years, the temperature has regularly touched 40 °C and above for days and in June 2024, Karachi experienced the hottest day of the year, with temperatures reaching 47.2°C.^28^ This study will be conducted in representative urban settlements in Karachi (Bilal Colony and Mehran Town), which primarily comprises mixed construction typologies as most buildings are RCC structures or girder and concrete tiles with load-bearing block masonry walls, while some lower-income strata have semi-permanent constructions using concrete blocks, mud bricks or corrugated metal sheets.

### Study design

**Figure 1:**
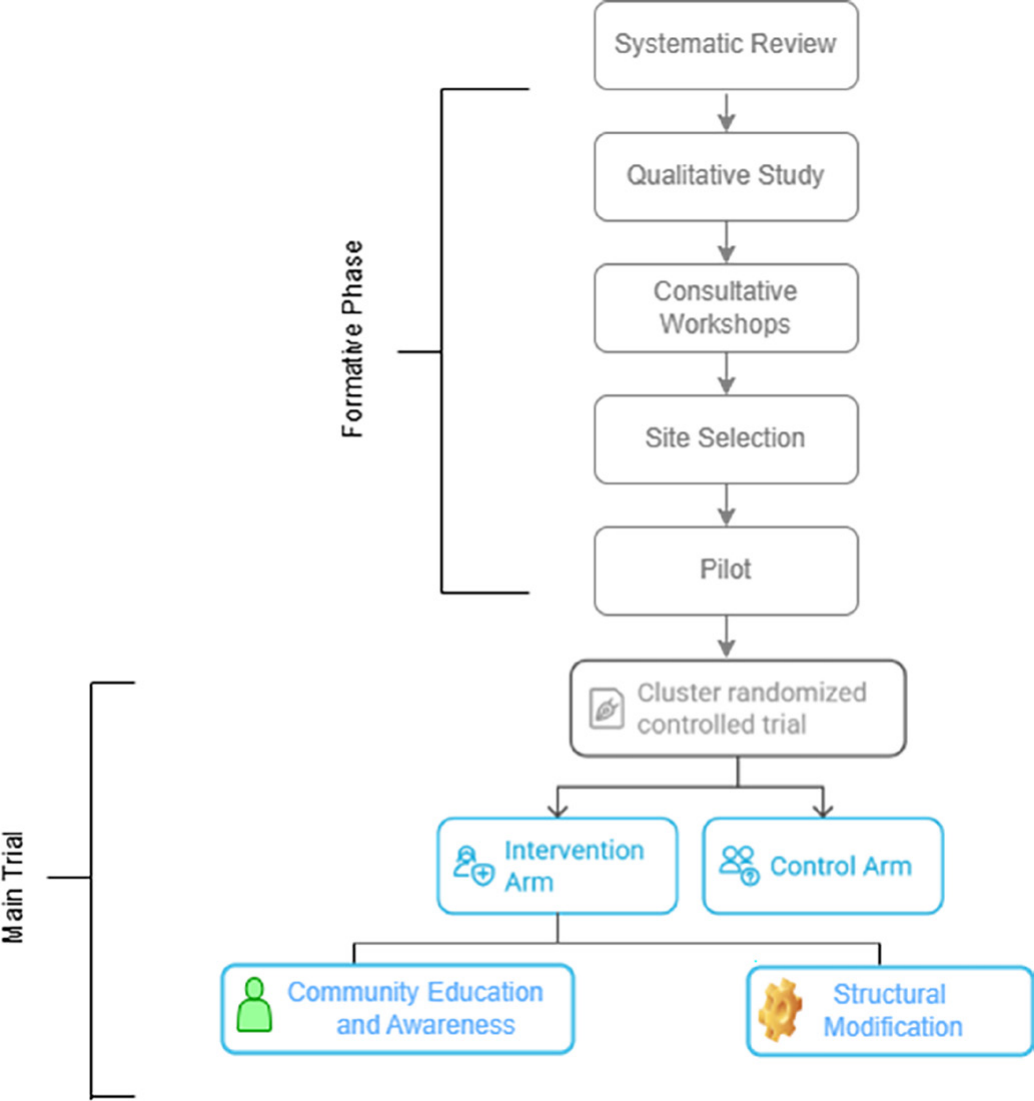
Study design diagram.

This study is a 2-year prospective, cluster randomised controlled trial designed to evaluate the effectiveness of ReHAB intervention in urban, low-income settings. Clusters are defined as localities comprising 70–80 households which typically span one to two contiguous streets, and these clusters will be randomly assigned to either the intervention or control arm in a 1:1 ratio (figure 1). Due to the nature of the intervention, blinding of participants and researchers will not be feasible, but data analysis would be blinded. The trial is registered on clinicaltrial.gov with trial registration number NCT06991543. The study is planned to start from 1 January 2026 to 31 December 2027.

### Population

The population of this trial will include all members of the households within the selected clusters who provide informed consent. A subset of eligible adults aged 18–60 years will be randomly selected for detailed physiological and personal heat exposure assessments.

#### Exclusion criteria

We will exclude any clusters that present significant accessibility or security concerns, have inadequate structural stability for retrofitting or have limited community cooperation. For the subset population, individuals unwilling to wear both (Fitbit and temp-u) devices simultaneously, individuals with cognitive or severe mental health conditions that may impair their ability to provide informed consent or comply with study procedures and individuals with severe physical disabilities that would limit participation in physiological monitoring will be excluded.

### Outcomes

**Figure 2:**
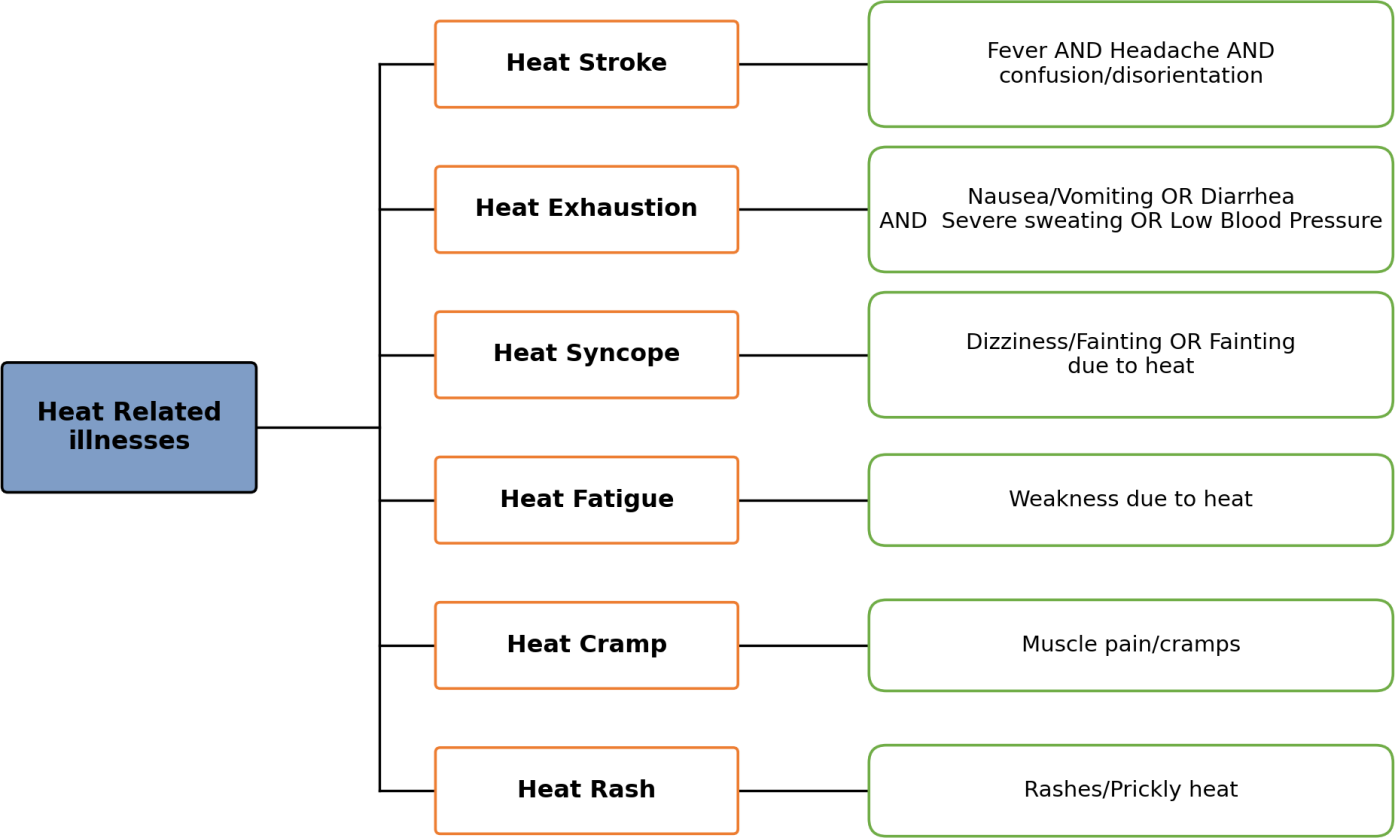
Categorization of structural interventions.

All primary, secondary, and outcomes assessed for the subset of the population, along with definitions, are provided in table 1 and figure 2.

**Table 1:** Outcome and definitions

Primary outcomes	
Heat-related illness	Defined as a documented heat condition/illness according to the International Classification of Disease (ICD-11) and institutional classification, which classifies heat-related illness as heat stroke, heat syncope, heat exhaustion, heat fatigue, heat cramps and heat rash ^37 38^(figure 2).
Quality of life	Defined as an individual’s perceived quality of life within the context of their culture, value systems, personal goals, expectations, standards and concerns.
**Secondary outcomes**	
Health outcomes	
All-cause mortality	Defined as the total number of deaths in the study population over the study period
Hospital visit/ hospitalisation	Defined as the number of hospital admissions (primary, secondary or tertiary facilities) during summers
Environmental outcomes	
Heat index	Indoor heat index (temperature and humidity) (^39^)
Universal Thermal Climate Index (UTCI)	Defined as a biometeorological index that describes the human body’s physiological response to the environment. It integrates air temperature, humidity, wind speed and radiant temperature to assess the overall thermal stress on the human body
Thermal comfort	Defined as change in the condition of mind that expresses satisfaction with the thermal environment
Outcomes measured for subset of population (Individual Level)	
Personalised heat exposure	Defined as mean daily external temperature each individual experiences (indoors and outdoors).
Heart rate	Defined as mean heart rate measurements (beats per minute) taken at 1-hour interval.
Sleep quality	Defined as a biological process that alternates between non-rapid eye movement (NREM) sleep and rapid eye movement (REM) sleep.
Physical activity	Defined as mean daily duration of sedentary, lightly active, moderately active and very active.
Energy expenditure	Defined as the average total number of calories burnt in a day.
Economic outcomes	
Cost effectiveness	Defined as the difference in cost divided by the difference in health effect between the intervention and control groups

### Sample size

Sample sizes were calculated for both the primary outcomes to provide at least 80% power at the 5% significance level. Based on heat-related symptoms and illness prevalence of 28.9%^29^ and an estimated intra-cluster correlation coefficient (ICC) of 0.0122, we calculate that 11 clusters per arm (22 clusters in total) with 70–80 households per cluster are needed to detect a 30% relative difference in heat-related illnesses while allowing for a 10% attrition rate. We treat 28.9% as a conservative estimate given the longitudinal nature of the primary outcome, as cumulative incidence over the surveillance period is expected to exceed this single-timepoint prevalence. To assess quality of life changes, we estimate that with 11 clusters per arm, 60 households per cluster are required based on the variability in baseline WHOQOL-BREF scores reported,^30^ which documented an average score of 68 (18.0) to detect a difference of 4 points in quality of life score, assuming an ICC of 0.015. As the heat-related illness outcome required the larger sample size, the final enrolment target was set at 70–80 households per cluster, which also provides adequate power for the quality-of-life outcome. To assess physiological heat strain, we calculated that a sub-sample of eight adults (aged 18 years–60 years) per cluster (balanced by gender) would be required to detect a 15% change in average heart rate among those who are exposed to heat stress, assuming a baseline of 66±12 bpm,^31^ an ICC of 0.1736 and 90% power. Given the complexity of the assessment process, we anticipate a higher attrition rate in this subset and have inflated the sample size by 30% to 10 individuals.

For binary outcomes, the ICC was derived from an assumed cluster-level prevalence variation of ±10%, with the coefficient of variation (CV) estimated from this range and the ICC subsequently calculated using the relationship ICC=k² × P/(1−P), where k is the assumed between-cluster CV of the prevalence, following Pagel *et al*.^32^ For continuous outcomes, the ICC was estimated as the ratio of between-cluster variance to total variance,^33^ with total variance sourced from reference studies and a minimum between-cluster difference of ±9 units assumed. Each co-primary outcome is independently powered at 80% or above. To control the family-wise type I error rate across the two co-primary outcomes, hypotheses will be tested using a pre-specified fixed-sequence (hierarchical) procedure: heat-related illness will be tested first at the two-sided 0.05 level, and quality of life will be tested at 0.05 only if the first test is significant. As each outcome is independently powered, this procedure incurs no loss of power. Quality of life is assessed at three time points per year over 2 years; the cross-sectional calculation above is therefore conservative, as the planned repeated-measures mixed-effects analysis increases precision by combining information across visits.

## Formative phase

The ReHAB intervention includes two core components: (1) community education and awareness and (2) structural heat adaptation modifications (figure 1). The formative phase comprised the following activities, which were undertaken to identify the specifics of the potential interventions which are plausible, acceptable and cost-effective.

### Systematic review

We conducted a systematic review of existing community-based interventions for heat adaptation in LMICs,^27^ analysing 185 studies and identifying effective strategies and highlighting gaps in adaptation strategies. Based on this information, we compiled a list of proven interventions to discuss with local communities and other stakeholders and policymakers.

### Qualitative study

The second part of this formative phase was in-depth interviews and focus group discussions with key stakeholders, including policymakers, relevant government officials at the national/provincial/local administrative level, civil society representatives, and community members, especially women, and the findings of this helped assess the possible education and structural interventions and their mode of delivery. The details of which would be published as a separate paper.

### Consultative workshops

A series of consultation meetings with key stakeholders, ranging from municipal authorities and related government departments and technical experts to community representatives, were held to finalise the intervention plans.

### Site selection

To identify the most feasible and representative location, a comprehensive site selection activity was undertaken across Karachi. A total of 30 potential sites were visited, with multiple visits conducted at each location. These visits involved assessing the built environment, understanding the local socio-economic dynamics, identifying climate-related vulnerabilities and gauging community willingness. Informal discussions were held at each site to capture local perspectives and ensure contextual relevance in site selection.

### Pilot study

A pilot study will be carried out in Karachi involving 80 households to assess implementation and recruitment feasibility, including participant recruitment and retention, acceptability of intervention delivery, adherence to wearable devices, and completeness of data collection procedures. Over the course of this pilot, the full suite of interventions will be delivered, community engagement activities will be implemented, and environmental data will be collected to assess their impact on indoor and outdoor temperatures; details will be published as separate papers. Once the pilot is concluded, the data will be analysed and reviewed in consultation with stakeholders and subject-matter experts. Findings from the pilot will inform refinement of intervention components and study implementation procedures and will not be included in the final trial analysis

## Intervention

### Intervention arm: Resilience and heat adaptation bundle

ReHAB was finalised based on the activities from the formative phase. The ReHAB consists of two components: community education and awareness and participatory structural modifications, which would provide local heat adaptation solutions. Behavioural and educational interventions will be delivered through community mobilisation whereas structural interventions will encompass modifications in the existing structures that communities live in to lower the indoor ambient temperature. Details of each component are provided below:

#### Community education and awareness

This component of the ReHAB will involve community mobilisation, which will include education and awareness activities throughout the intervention duration. Community groups (CGs) will be established in all the 11 intervention clusters with separate male and female representatives working as volunteers. The CGs will be responsible for community mobilisation activities and surveillance for the primary outcome (heat illness). Each CG will comprise 6–8 members, representing a diverse group of people, including local government members, local elders, religious leaders and prominent male and female members of the community. These CGs will facilitate dissemination of early warnings regarding heat waves, awareness sessions and door-to-door outreach focused on heat-health fundamentals, hydration practices, appropriate clothing and daily activity scheduling. CGs will also maintain simple logbooks to track any visit to a healthcare facility within their catchment area.

Also building on this CG platform, hands-on demonstration and reinforcement of specific cooling behaviours will be emphasised. CG members, along with the study team, will lead small-group demos on practical techniques, such as setting up shade cloths, using light-coloured clothing, wet-towel wraps, misting fans, changing work timings and following work-rest-shade cycles to reduce heat exposure. CGs will also distribute the relevant information, education and communication (IEC) material. This structured, behaviour-focused delivery aims to ensure both the uptake and sustainability of protective cooling practices. Community hydration arrangements, like setting up temporary drinking water facilities (‘Sabeels’), will also be put in place as part of the intervention package to reduce dehydration among outdoor workers.

#### Structural modification

**Figure 3:**
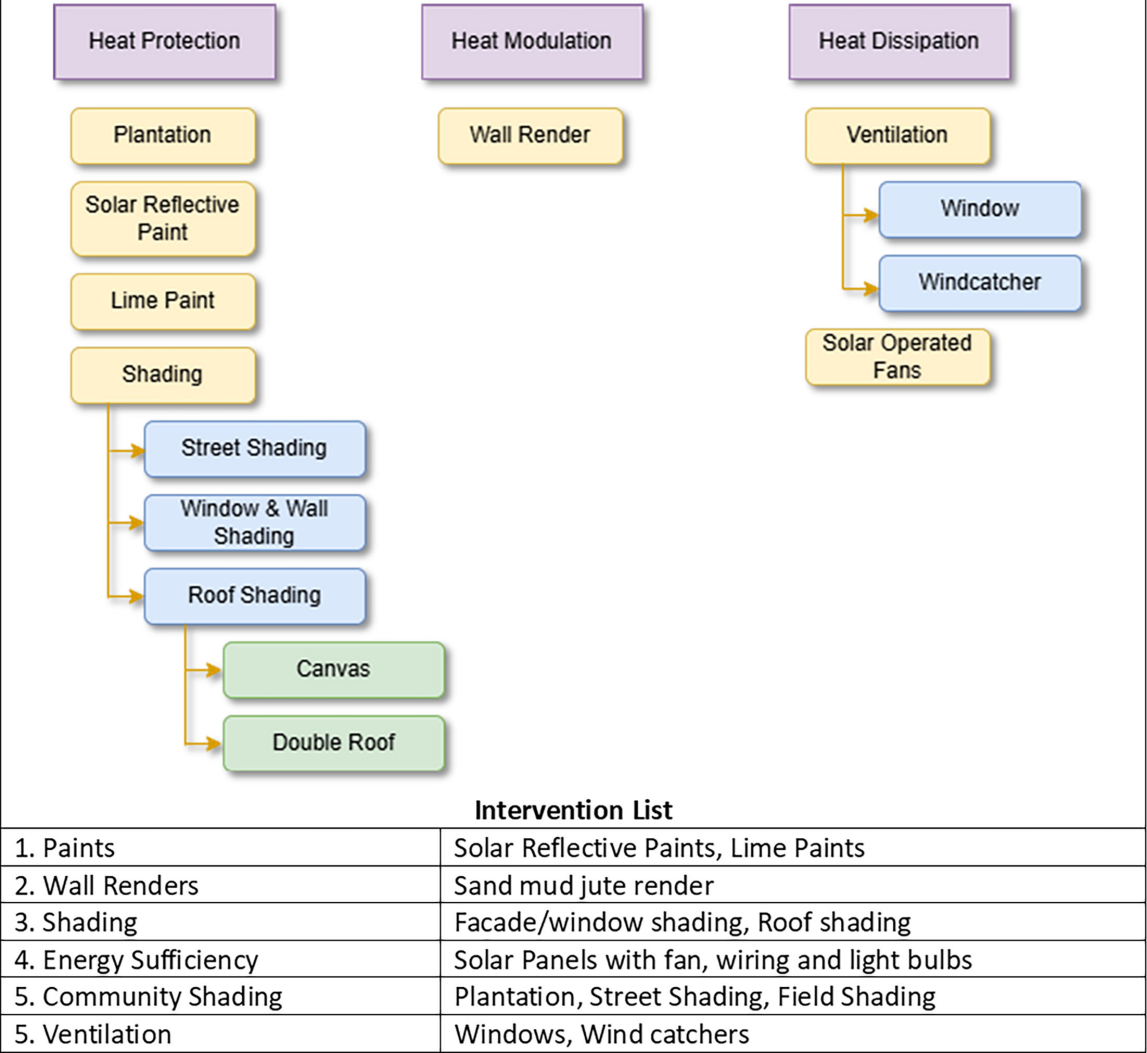
Building survey and intervention process.

**Figure 4:**
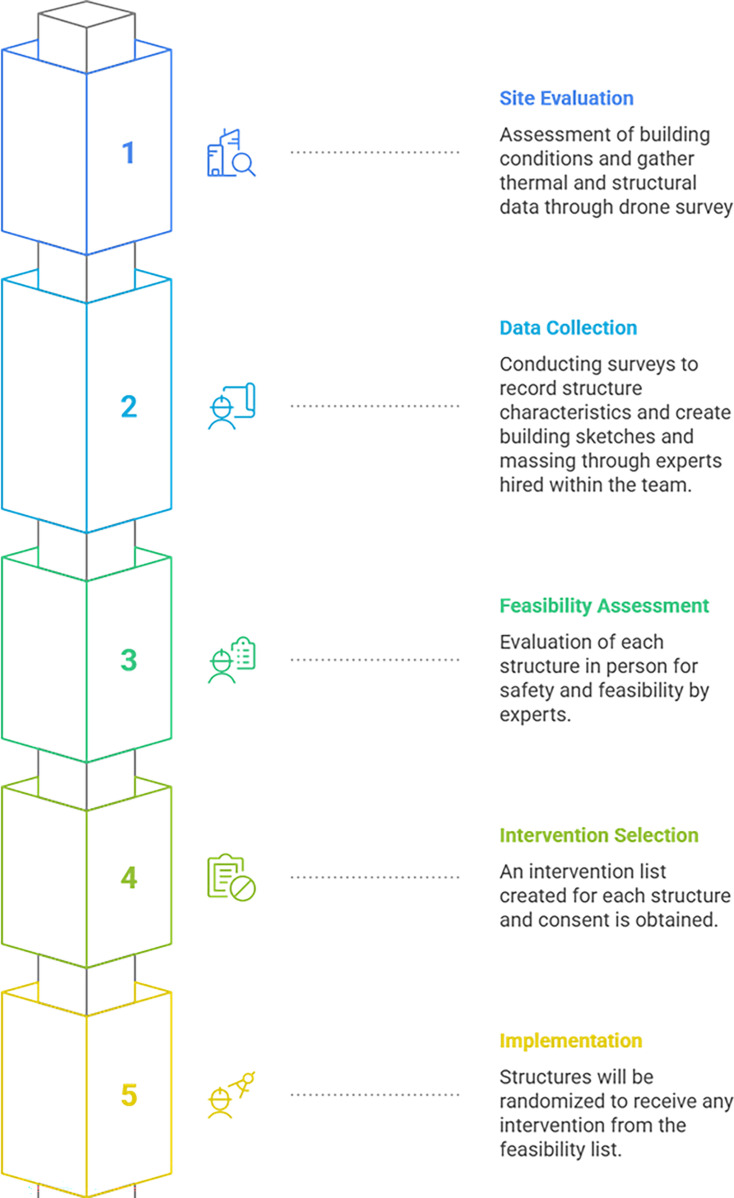
Classification of Heat Illness Symptoms.

This component will include modifications to existing structures (retrofitting) and community spaces. The specific structural intervention considered for each household within a cluster and the communal intervention will be selected from the most feasible interventions identified in the formative phase (figure 3). The household-level interventions are targeted to reduce indoor temperature, and the community interventions will play a role in reducing the effects of high heat for outdoor workers and the community. The selected interventions based on the extensive formative work are divided into three categories: heat protection, heat modulation and heat dissipation.^34^ Intervention for each selected household/structure and community space within the intervention cluster will be evaluated through a series of steps including building materials used, strength, efficiency, overshadowing, ventilation conditions and consent of residents, and after assessment of structural safety and resident safety by experts including architects and structural engineers, intervention for each dwelling would be finalised and delivered (figure 4). We will make every effort to ensure that each individual intervention is delivered proportionately in each cluster, that is, an equal number of each intervention type in every cluster.

These interventions will be delivered based on a community-participatory approach; the community and the project will share the cost of intervention to improve ownership, as shown in a previous trial.^35^ The community contribution will be approximately 15–25% of the total cost; this contribution can be monetary or non-monetary (labour). This approach would enhance the acceptability and sustainability of the interventions.

##### Information, education and communication materials

Findings from the formative phase will guide the development of educational materials. We will develop the following materials.

- Posters.
- Pictorial brochures/flip charts.
- Short video messages in the local languages (Sindhi, Pashto and Urdu).

IEC materials will be distributed during CG meetings and during follow-ups through data collectors, and short videos will be shared through WhatsApp groups with disappearing message settings turned on (messages that disappear after a single view) to prevent videos from passing on and possibly resulting in control group contamination.

##### Training

All CGs will be trained in specific awareness and educational messages regarding heat prevention and adaptation measures to assist them in carrying out the sessions. Along with this, all CGs will be trained in the care of temperature devices, Android devices and data loggers and establishing a network within the community for effective surveillance of heat-related illness and hospital visits. The CGs will also be trained to effectively communicate with the community about structural intervention decisions and explain the reason for selecting a particular intervention for a structure.

##### Sessions

The community sessions will be led by CGs and held separately for males and females. It will consist of:

- CG meetings (triggering session).
- Meetings supervised by research staff.

The sessions will focus on educating the community. The meeting schedule will be as follows:

- Summer (April–June):
  - Fortnightly community meetings.
  - Weekly WhatsApp video messages.
  - Weekly weather alerts.
- Late Summer (July–October)
  - Monthly community meetings.
  - Weekly WhatsApp video messages.
  - Weekly weather alerts.
- Winter (November–March):
  - Community meetings every other month.
  - No WhatsApp video messages.

### Control arm

The control arm of the study will not receive any intervention other than routine care and information available through existing government response systems, including health and disaster management agencies.

## Data collection

Data collectors will undergo comprehensive training over a week to ensure a clear understanding of data collection tools, operational protocols, methodologies and management procedures. To assess their comprehension, they will complete practice questionnaires in the field and will also be trained in device and data management by experts.

### Line listing

The line listing and cluster assessment process is intended to establish a complete sampling frame for eligible clusters. A census of households will be done in the whole Bilal colony and Mehran town. The data collected will include household ID; number of floors in a structure (including basements, if any); total number of members, elderly, children and women of reproductive age (WRA) within a household; years lived within the same structure and primary material of floor and roof and wall. After the line listing, clusters of 90–120 households will be assigned a number by combining two to three streets depending on the number of households present in each street, which would result in approximately 300 clusters. We will then exclude clusters with inaccessibility or security concerns and for feasibility in terms of community cooperation and structural integrity for the proposed retrofitting interventions and the remaining clusters would then be randomised in the intervention and control groups.

### Cluster randomisation

We will employ a constrained randomisation procedure as described by Lock, Morgan and Rubin (2012),^36^ rather than relying on a single random allocation, the randomisation process will be implemented using the Stata ‘randomise’ command, which generates multiple allocation schemes in the background and evaluates balance across prespecified baseline covariates. An allocation achieving an acceptable level of balance between study arms at the cluster level will then be selected for the trial. Geographically designed clusters will be randomly assigned by an independent statistician to either the intervention arm, receiving the Heat Adaptation Bundle (ReHAB) or the control arm. Each random allocation is evaluated for covariate balance on three pre-specified characteristics: population residential density, household structural type (single-storey vs double-storey) and roof type (permanent, semi-permanent or natural materials).

Additionally, simple randomisation would be used to select eight households for the in-depth assessment from a subset in both intervention and control groups. Within these selected households, the Kish grid method would be used to randomly select the eligible adult if more than one adult is present. If the indexed participant refuses, the next eligible adult will be considered from the same household. If all eligible adults from a household refuse to participate, then another household will be included. Male and female participants for the subset will be selected systematically.

### Household data collection

Baseline, monthly/weekly follow-up and end line surveys will be conducted in 22 randomised intervention and control clusters. Baseline and endline surveys will be done to collect information on various variables, including demographics (household size, socioeconomic status, education, occupation, ethnicity), healthcare seeking, mental health using the WHO wellness survey, food security, dietary diversity, co-morbidities, medications, signs and symptoms of heat-related illness (box 1). The questionnaire was developed using standardised questionnaires used during similar surveys, and some questions were modified to better accommodate local contexts. The tool was then translated into the local language and back-translated to English to validate the translation.

Box 1 **Section for data collection tool**

**Description of modules and their sections**

Module-A: Household Identification and Demographic Information

- Section-A1: Household Identification and Consent
- Section-A2: Household Members’ Information
- Section-A3: Socioeconomic Status of Household (SES)
- Section-A4: Building Survey
- Section-A5: Household Water and Sanitation Status

Module-B: Household Food Insecurity, Dietary Diversity and Source Assessment

- Section-B1: Household Food Insecurity Experience Scale
- Section-B2: Household Dietary Diversity and Source Assessment

Module-C: Health and Hygiene Assessment of Adult Participants

- Section-C1: General Health and Disabilities
- Section-C2: Mental Health
- Section-C3: Hygiene Practices (Hand Washing, Dental Hygiene, Bath, (Menstrual Hygiene for Female)

Module-D: Heat Exposure/Sleep/Dietary Assessment

- Section D1: Work and Sun Exposure
- Section D2: At-home heat exposure
- Section D3: Heat Stress Experiences, Food Security and Adaptation Strategies
- Section D4: Knowledge About Heat-Related Health Problems, Existing Heat Adaptation and Prevention Strategies
- Section-D5: Sleep
- Section-D6: Quality of life

Data on the signs and symptoms of heat-related illness will be collected through multiple points to ensure no event is missed during the study period. Data collectors will conduct weekly household visits to collect symptom data. Events identified through community surveillance will trigger a household visit by data collectors who will use the same survey tool, and reported health facility visits will be verified through facility records where feasible. Symptoms will be classified as International Classification of Disease-11 and institutional classification of heat illness (figure 2). Data for quality of life will be acquired through multiple surveys of all households included and the respondent for these surveys will be the selected index adult (table 3).

Additionally, at endline, 5 mL of venous blood samples will be collected by trained phlebotomists using standard aseptic techniques from control and intervention households during peak heat in year two of the project. These samples will be stored for further evaluation in our biobank following standard storage guidelines. They may be used to detect heat-related biomarkers in any future research.

Quantitative data collection will be conducted using handheld devices equipped with a customised application developed in Java, using MySQL and SQLite for data storage. The programme will incorporate range and consistency checks, along with skip patterns to reduce data entry errors. Any detected inconsistencies will be addressed by contacting data collectors for verification and correction. Each participant will be assigned a unique code generated by the Android application to ensure anonymity. All data will be securely stored behind firewalls and fully anonymised before analysis.

### Environmental data collection

Environmental data collection will include a thermal drone survey to assess the comparative heat accumulation and dissemination of each structure. A drone survey of each cluster will be conducted before intervention and once yearly at three points, that is, at sunrise (within 2 hours), mid-day (between 12:00 and 16:00) and sunset (1 hour before sunset and 1 hour after sunset).

Indoor temperatures of all households included will be recorded using thermochron iButtons set to record temperature at 20-min intervals. Outdoor temperatures and humidity will be recorded using HOBO weather stations installed at each site and Kestrel drop devices (also recording at a 20-min interval). Additionally, local weather station data will be used to obtain daily information on ambient temperature, relative humidity, wind speed and solar radiation throughout the study period. Heat stress will be calculated by integrating these parameters to formulate heat indices, including the Universal Thermal Climate Index (UTCI) and the Wet Bulb Globe Temperature.

Designated personnel will regularly retrieve environmental data from wearable and installed devices, returning them to participants after each data transfer. In areas with limited internet access, the team leader will manually export the data onto a USB device and store it securely on a password-protected laptop to prevent data loss. All data will be archived in a secure repository at Aga Khan University (AKU), Karachi, with access restricted to authorised data management personnel through AKU-LAN identification. Role-based access permissions will be implemented to ensure confidentiality and data security. To prevent data loss or corruption, the repository will be synchronised daily with a secure remote backup system.

### Devices

The devices and their utilisation for outcome measurements are provided in table 2:

**Table 2:** Devices used for outcome measures

Device	Outcome measures	Placement
Thermochron ibuttons, Temp-U, which record temperature and humidity	Individual and household-level exposure	Put it at select households in the intervention and control clusters within the main living room, midway between floor and ceiling and away from any direct heat source. Devices will be given to individuals selected for the sub-group for individual exposure assessment
Kestrel 5400, which will record multiple environmental parameters like wet bulb globe temperature (WBGT), solar radiation, barometric pressure, wind speed and humidity	Environmental exposure	One device rotated in each cluster
Fitbit Inspire 3 is a wearable fitness tracker that will track heart rate, sleep cycles, activity levels and energy consumption	Individual physiological outcomes	Given to the sub-set population selected in the intervention and control clusters
Kestrel drop D2 and HOBO U-30 weather stations	Weather	One weather station placed at the field office as a central location and one Kestrel drop D2 placed in each cluster

### Data collection from subset population

Monthly data will be collected for in-depth understanding of heat stress and intervention effectiveness within the subset population (table 3). This will include thermal comfort, mental health and heat exposure assessment.

**Table 3:** Schedule of outcome assessments and follow-up activities

Parameter	Time points	Details	Devices/Tools	Population/Unit
Primary outcomes				
All heat-related illness	Weekly during peak summers	Heat-related illnesses	Weekly community survey, health facility data, community surveillance	All members of included households
Quality of life	Mid of April, July and December for two consecutive years	Quality of life mean score	WHOQOL-BREF* questionnaire	Indexed adults of all included households
Secondary outcomes				
All-cause mortality	Weekly during peak summers	Any mortality occurring within the included clusters	Weekly community survey, health facility data, community surveillance	All members of included households
Hospital visit/hospitalisation	Weekly during peak summers	Any hospital admission or hospital visit occurring within the included clusters	Weekly community survey, health facility data, community surveillance	All members of included households
Indoor heat index	Continuous throughout the year	Indoor data loggers	iButton, Kestrel Drop D2, Temp-U	All included households
Thermal comfort (home)	Monthly	Self-reported comfort levels	Thermal comfort survey adapted from CHEQ-5†	Indexed adults randomised for sub-group
UTCI§	Continuous throughout summers	Outdoor only	Kestrel Drop D2, Kestrel 5400, HOBO weather station	Overall catchment area
Outcomes measured for subset of population				
Personalised heat exposure	Baseline, monthly	Occupation, sun exposure, illness history, daily routine	Structured survey tool (adapted from HotHaps)‡ and Temp-U	Indexed adults randomised for sub-group
Physiological strain markers	Continuous throughout summers	Heart rate, sleep cycles, VO₂¶ max	Fitbit Inspire 3	Indexed adults randomised for sub-group

* WHOQOL-BREF: World Health Organization Quality of Life - Brief

† Cold and Heat Exposure Questionnaire

‡ High Occupational Temperature Health and Productivity Suppression

§ Universal Thermal Climate Index

¶ Volume of Oxygen

Individual heat exposure of a subset of participants (eight per cluster) will be measured by attaching a thermochron ibutton or Temp-U to their clothing. The participants will be instructed to always wear this logger. At the time of sleeping, they will be instructed to place the logger on their bedside.

The participants from the sub-set will be provided with Fitbit Inspire 3 devices to record physiological markers: heart rate, daily body temperature change, sleep, energy expenditure and activity. These smartwatches will be synced to the AKU server weekly and returned to the participants after charging.

Participant compliance will be monitored through spot checks and periodic review of device data. For Fitbit devices, heart rate data will be reviewed, as prolonged absence of heart rate recordings may indicate that the device was not worn. Similarly, iButton and Temp-U data will be checked periodically for implausible temperature spikes or sudden drops, which may indicate non-compliance or incorrect device use.

### Statistical analysis

The trial has two co-primary outcomes, heat-related illness and quality of life. These outcomes will be evaluated independently to assess the effectiveness of the intervention. All statistical analyses will be conducted using STATA 18.0 (https://www.stata.com), following an intention-to-treat approach while accounting for the cluster-randomised trial design. Baseline characteristics will be analysed to assess comparability between intervention and control arms. Descriptive statistics will summarise household and participant characteristics, and any important imbalances at baseline will be adjusted for in the analysis. No interim analysis or formal stopping guidelines are planned given the low-risk nature of the intervention.

Statistical analyses will account for the cluster-randomised design by incorporating clustering through random effects or robust variance estimators, as appropriate. For outcomes measured repeatedly over time, within-participant correlation will be accommodated using repeated-measures mixed-effects models. Intervention assignment and seasonal variation will be included as fixed effects in all applicable models, while relevant baseline covariates and baseline outcome values (where available) will be adjusted for. Model assumptions, including overdispersion for count outcomes, will be assessed and alternative modelling approaches (eg, negative binomial models) will be used where appropriate. Missing data will be examined and handled using complete-case analyses or multiple imputations, depending on the extent and pattern of missingness. Sensitivity analyses will be performed to assess the robustness of findings to different assumptions regarding missing data.

#### Primary outcome analysis

For each participant, a summary record will be constructed comprising the number of heat-related illness episodes and the number of assessment occasions (or person-time) over which the individual was observed. To assess the intervention effect, a Poisson regression model with cluster-level random intercepts will be used, accounting for intra-cluster correlation. The number of heat-related illness episodes/events will serve as the numerator, while person-weeks at risk will be used as the denominator. Results will be presented as incidence rate ratios (IRRs) with 95% CIs.

Duplicate reports identified across multiple sources will be flagged in the database using participant ID, household ID, date of onset, symptoms and care-seeking information to ensure that each event is counted only once. Symptoms or events continuing from a previous reporting period will be considered part of the same illness episode. A new episode will be defined as either the onset of symptoms following a symptom-free interval of at least two days after the previous episode or the occurrence of additional new symptoms in a participant with ongoing previously reported symptoms. For quality-of-life assessments, the mean increase from year 1 to year 2 would be compared three times a year. Summer and winter quality of life means would be segregated and compared. Results will be reported as adjusted mean differences with 95% CIs.

#### Secondary outcome analysis

Secondary outcomes will be analysed within the same cluster randomised framework, accounting for clustering at the design level using mixed-effects models or generalised estimating equations as appropriate to the distribution of each outcome. All-cause mortality and hospital visits/hospitalisations, measured as counts or rare events, will be analysed using mixed-effects Poisson or logistic models (or Cox proportional hazards models where individual-level time-to-event data are available), with results reported as IRRs, risk ratios or HRs with 95% CIs. Indoor heat index and UTCI, both continuous environmental exposure measures, will be assessed using linear mixed-effects models with random intercepts for clusters and adjustment for temporal variation to estimate mean differences between trial arms. Thermal comfort, measured either as a continuous score or ordinal response, will be analysed using linear mixed models or ordinal logistic mixed-effects models depending on scale properties. Subgroup analyses will be conducted by including interaction terms between intervention assignment and key effect modifiers such as age (WRA, children and elderly), sex and occupational exposure. All models will be adjusted for relevant covariates where appropriate, and robustness will be assessed through sensitivity analyses including alternative correlation structures. Cost-effectiveness analysis will be conducted to evaluate the economic feasibility of different adaptation strategies.

Analysis for sub-set outcomes measured in the subset of the population will be analysed using multilevel mixed-effects models to account for repeated measurements within individuals and clustering at the household or cluster level, as appropriate. Personalised heat exposure, collected longitudinally, will be analysed as a continuous outcome using linear mixed-effects models incorporating time-varying covariates such as occupation, daily activity patterns and reported illness history. Physiological strain markers obtained from wearable devices (eg, heart rate, sleep metrics and related indices) will be analysed using hierarchical mixed-effects models to estimate intervention effects on both average levels and temporal trajectories over the study period, with additional smoothing or functional approaches considered for high-frequency data. All analyses will report adjusted mean differences with corresponding 95% CIs and will explore heterogeneity in effects over time and across participant characteristics where data permit.

### Ethical considerations

This study will be conducted under strict ethical guidelines to ensure the safety, dignity and rights of all participants. Ethical approvals were obtained from the Ethical Review Committee of the Aga Khan University (ref: 2025–11548-35626) and the National Bioethics Committee (ref: 4–87/NBCR-1065/23/1736). All household and community gatekeepers will provide written informed consent, and for those with literacy challenges, an oral consent option will be available, recorded with a signature or thumb impression in the presence of a witness (online supplemental file 1). Participants will be fully informed about the study’s objectives, procedures, potential risks and benefits. Separate consent will be obtained for physiological monitoring through wearable devices and for structural modifications.

A separate, explicit consent process will explain the purpose of blood sample collection, the volume of blood drawn, potential laboratory analyses, storage duration and whether samples may be used for future ethically approved research. Participants will be informed of minimal risks associated with venipuncture, including mild pain, bruising, dizziness or rare infection and appropriate clinical precautions will be taken to minimise these risks. Samples will be processed and stored for at least 7 years in a secure, access-controlled biobank facility compliant with national regulatory standards. All specimens will be de-identified and labelled with unique study identification codes. Personal identifiers will be stored separately from biological samples in password-protected databases accessible only to authorised study personnel. Participants will retain the right to withdraw consent for storage and request destruction of their samples at any time.

Confidentiality will be maintained by anonymising all participant data using unique study IDs. Personal identifiers will be securely stored in a separate database, and data will be collected on encrypted electronic devices and stored on password-protected servers at AKU, with restricted access to authorised personnel only. Information will not be shared with third parties without consent, and results will be presented in aggregate form to prevent identification of individuals. Any potential discomfort from wearable devices or thermal exposure monitoring will be assessed, and participants will have the option to withdraw. Structural modifications and environmental interventions will be implemented in collaboration with local communities to avoid physical harm or undue burden.

Efforts will be made to ensure the equitable participation of marginalised groups, including women, the elderly and outdoor labourers. Community mobilisation activities will engage both male and female participants, and information will be provided in multiple languages, including Sindhi, Pashto and Urdu, to accommodate linguistic diversity. Findings will be shared with community members and policymakers to foster long-term sustainable adaptation strategies. Structural modifications and educational interventions will be co-developed with communities to enhance relevance and ownership.

Any ethical breaches will be reported, and corrective actions will be taken immediately. The study is committed to upholding the highest standards of research integrity while ensuring participant safety and well-being.

## Supplementary material

10.1136/bmjopen-2026-120115 online supplemental file 1
