# Supplementary material for: Structural heat adaptation and education in urban settings (SHAPES trial): study protocol for a cluster randomised controlled trial
Source: BMJ Open. 2026 Aug 11;16(8):e120115. doi: 10.1136/bmjopen-2026-120115 (PMC13475081; doi:10.1136/bmjopen-2026-120115)
Supplement: Supplementary file 1 [file bmjopen-2026-120115.reviewer_comments.pdf]

PEER REVIEW HISTORY

BMJ Open publishes all reviews undertaken for accepted manuscripts. Reviewers are asked to complete a checklist review form and are provided with free text boxes to elaborate on their assessment. These free text comments are reproduced below.

ARTICLE DETAILS

Title (Provisional)

Structural Heat Adaptation and Education in Urban Settings (SHAPES Trial): Study Protocol for a Cluster Randomized Controlled Trial

Authors

Das, Jai K.; Naqvi, Syeda Kanza; Bonell, Ana; burman, christopher; Augustin, Joseph; Qamar, Shahmeer; Rizvi, Arjumand; Chauhdary, Imran Ahmed; Naqvi, Anjum Abbas; Jamali, Muhammad Khan; Naqvi, Hassan; Tabassum, Farhana; Cousens, Simon; Bhutta, Zulfiqar A

VERSION 1 - REVIEW

**Reviewer**	**1**
**Name**	**Herrmann, Alina**
**Affiliation**	**UniversitatsKlinikum Heidelberg, Institute for Public Health**
**Date**	**13-May-2026**
**COI**	**None**

The SHAPE trial is a very timely study, as there is a great need for clear evidence of the effectiveness of climate change adaptation measures, especially in vulnerable communities like urban Pakistan, where prior heatwaves have been costing thousands of lives. The study protocol is well structured and reports transparently and in detail about all study procedures from recruitment to analysis. Below you can find a few points, were further clarification would be helpful.

Specific comments:

Box: Strength and limitations of this study

• Please define both aspects of “the adaptation measures”, possibly as “a bundle of educational and structural adaptation measures” as it is not necessarily the first trial testing structural changes (there are existing cool roof trials).

Background

• It is interesting and important for the presented trial, that the building structured is described in detail infrastructure in vulnerable neighborhoods the described concrete

infrastructure or is there also in. Later in the manuscript you write, that the there is also informal housing with less “dense” infrastructure than concrete buildings. Maybe you want to mention, that in a half sentence already in the background. Otherwise the reader might be surprised later on.

• Razzak et al. the citation numbers seem to have gotten mixed up, as Razzak et al. is cited as 28 in the text and 29 in the reference list.

Methods:

• In the primary objectives the assessment of feasibility is also mentioned. However, later on in the protocol, I have not seen the description of assessment of any feasibility or process-oriented outcomes. If there will be a feasibility assessment other than the pilot, you could say so and also make the feasibility objective a separate one. If only the pilot looks at the feasibility, please define the feasibility criteria a bit more specifically.

Outcomes.

• Throughout the paper there are several mentions about how heat related illness will be measured (e.g. Table 3 and data collection). Still, it doesn’t become entirely clear, how the outcome will be put together from the three tools (community survey, health facility data, community surveillance). As this is one of the primary outcomes, this is important to understand. Will health facility data be accessed for each participant at the end of the trial? Is the reporting of community members to CG-leads community surveillance, and if so, what is the additional weekly community survey? How will data from these three sources be combined (simple counting of incidences from different sources? What if the incidences in different data sources refer to the same incident?)

• In box 1 sometimes the concrete tools for outcome measurement are mentioned (e.g. quality of life) and sometimes not (e.g. thermal comfort). While all tools are mentioned in table 3, box one should be consistent by either mentioning all tools or none of them and referring to table 3 for outcome measurement tools.

• As table 2 refers to the devices rather than to the outcome measures, I would recommend to put the device in the first column and the outcome measure in the second.

• Personally I would find it more helpful if table 3 was inserted at an earlier stage, maybe closely after the objectives.

• An assessments of 300 possible clusters seems much, if only 22 are needed and you already seem to know which areas might be feasible (if I understood your formative work correctly). Could you explain, why you are taking a full census of the two neighborhoods? This doesn’t seem to fit the data protection principle of “data minimization” (only assessing as much data as necessary for the study purpose).

Primary outcome analysis:

• In the first sentence, it is only mentioned how morbidity is assessed, not mortality, although it is a combined outcome.

• Minor: three points or three times: Currently for quality of life it sais “three-points times”

• Please cite the reference for the “modified behavioural adaptation model”

Other issues:

• Will the data of the pilot be included in the analysis of the final study or only be used separately at the beginning?

• It is clear, that blinding is not possible in this trial. However, as the primary outcome (heat-related illness) is documented by community members, there seems to be a particular risk of observer bias. Could you discuss, how you could minimize this risk of bias?

• As the training and ongoing work of community group leaders is so extensice, do they receive and salary or incentive?

• In the CONSORT Checklist, there are a few items, that are identified as not applicable, but that are actually addressed in the protocol, for example 3 protocol (the study protocol is registered.

VERSION 1 - AUTHOR RESPONSE

No.	Source	Section / issue	Comment	Response / action taken
9	Reviewer 1	Strengths and limitations	Please define both aspects of “the adaptation measures”, possibly as “a bundle of educational and structural adaptation measures” as it is not necessarily the first trial testing structural changes (there are existing cool roof trials).	Response: We thank the reviewer for noticing this gap. We have now revised the section to explicitly say a bundle of interventions.<br><br>Manuscript changes: Page 2, under the heading “Strengths and limitations of this study”
10	Reviewer 1	Background	Clarify the description of building structures/infrastructure in vulnerable neighborhoods. The manuscript later mentions informal housing with less dense infrastructure than concrete buildings; consider mentioning this briefly in the background so the reader is not surprised later.	Response: Thank you for this helpful suggestion. We have revised the Background section to briefly describe the diversity of housing typologies commonly found in low-income urban settlements<br><br>Manuscript changes: Page 3, under the heading “Background”.
11	Reviewer 1	References	Razzak et al. citation numbers appear mixed up, as Razzak et al. is cited as 28 in the text and 29 in the reference list.	Response: Thankyou for pointing this inconsistency. We have now corrected this.<br><br>Manuscript changes: Page 3, under the heading “Background”.

12	Reviewer 1	Methods: objectives/feasibility	In the primary objectives, assessment of feasibility is mentioned. However, the protocol does not clearly describe assessment of feasibility or process-oriented outcomes. If feasibility will be assessed beyond the pilot, state this and make feasibility a separate objective. If only the pilot assesses feasibility, define feasibility criteria more specifically.	Response: Thank you for this important comment. Feasibility assessment forms part of an embedded pilot phase within the main trial rather than a separate preliminary study. We have revised the manuscript to clarify this and have added details regarding the aspects of feasibility being assessed.<br><br>Manuscript changes: Page 7, under the heading "Pilot Study".
13	Reviewer 1	Outcomes: heat-related illness	Clarify how heat-related illness will be measured and constructed from the three tools: community survey, health facility data, and community surveillance. Explain whether health facility data will be accessed for each participant, what community surveillance entails, how it differs from the weekly community survey, and how duplicate incidents across sources will be handled.	Response: Thank you for this important comment. We have revised the manuscript to clarify how heat-related illness events will be identified and constructed across the three data sources. We have also clarified the process for identifying duplicate reports across sources and added a definition for distinguishing continued illness episodes from new events.<br><br>Manuscript changes:<br>Identification of heat-related illness = Page 12, under the heading "Household Data Collection.<br>Identifying duplicates = Page 15, under the heading "Statistical Analysis".
14	Reviewer 1	Outcomes: Box 1	In Box 1, outcome measurement tools are sometimes mentioned (e.g., quality of life) and sometimes not (e.g., thermal comfort). Make Box 1 consistent by either mentioning all tools or none, and refer to Table 3 for outcome measurement tools.	Response: Thank you for this comment. We agree that Box 1 should maintain a consistent structure. We have revised Box 1 to focus only on outcome definitions and removed references to specific measurement tools. Details regarding measurement tools and assessment methods are now consistently presented in Table 3.<br><br>Manuscript changes: Page 5-6, Box 1.
15	Reviewer 1	Table 2	As Table 2 refers to devices rather than outcome measures, put the device in the first column and the outcome measure in the second column.	Response: Thank you for this suggestion. We have revised the table so that the first column lists the devices, and the second column specifies the corresponding outcome measures.<br><br>Manuscript changes: Page 13, Table 2.

16	Reviewer 1	Table placement	Consider inserting Table 3 earlier in the manuscript, perhaps close to the objectives.	Response: Thank you for this suggestion. We appreciate the rationale for positioning Table 3 closer to the objectives. However, we believe its current placement within the data collection section maintains the logical flow of the manuscript, as the table contains detailed information regarding outcome assessment methods, timing, devices, and study populations that are closely linked to data collection procedures. We have therefore retained its current position for readability and contextual consistency.<br><br>Manuscript changes: None.
17	Reviewer 1	Cluster assessment / data minimization	Explain why a full census/assessment of 300 possible clusters is needed when only 22 clusters are required and the feasible areas seem already known from formative work. The reviewer notes this may not fit the data protection principle of data minimization.	Response: Thank you for this important comment. The purpose of the line listing and assessment of approximately 300 potential clusters is not to enroll all clusters, but to establish a complete sampling frame of eligible areas for randomization. Only limited information necessary for cluster identification and eligibility assessment will be collected, in accordance with data minimization principles. We have clarified this in the manuscript.<br><br>Manuscript changes: Page 10, under the heading “Line Listing”.
18	Reviewer 1	Primary outcome analysis	In the first sentence, only morbidity is described, not mortality, although the primary outcome is combined morbidity and mortality.	Response: Thank you for identifying this inconsistency. We have revised the primary outcome analysis section to clarify that the primary outcome includes only heat-related morbidity while mortality remains a secondary outcome.<br><br>Manuscript changes: Page 15, Under the heading “Primary outcome analysis”.
19	Reviewer 1	Minor wording	Correct the phrase “three-points times” for quality of life; clarify whether it should be “three points” or “three times.”	Response: Thankyou for pointing this out. We have now corrected the phrase.<br><br>Manuscript changes: Page 15, under the heading “primary outcome analysis”.

20	Reviewer 1	Reference needed	Please cite the reference for the "modified behavioural adaptation model."	Response: Thankyou for your comment. We have modified the statistical analysis section of the protocol.<br><br>Manuscript changes: Page 15-16, under the heading "Statistical analysis"
21	Reviewer 1	Pilot data	Clarify whether pilot data will be included in the final study analysis or used separately only at the beginning.	Response: Thank you for this comment. We have clarified in the manuscript that data generated during the pilot phase will be used only to assess and refine intervention and implementation procedures and will not be included in the final study analysis.<br><br>Manuscript changes: Page 8, under the heading "Pilot phase".
22	Reviewer 1	Risk of observer bias	Discuss how risk of observer bias will be minimized, since blinding is not possible and the primary outcome (heat-related illness) is documented by community members.	Response: Thank you for this important comment. We acknowledge the potential for observer bias given that participant and field team blinding is not feasible. To address this, we have clarified within the existing methods sections that community group members will only identify and notify potential events rather than classify heat-related illness. All reported events will be assessed using the same standardized survey tool and predefined criteria by trained data collectors, health facility records will be used for verification where available, duplicate events will be reconciled across sources, and outcome analysis will remain blinded to allocation status.<br><br>Manuscript changes: Page 12, under the heading "Household data collection".
23	Reviewer 1	Community group leaders	Clarify whether community group leaders receive a salary or incentive, given the extensive training and ongoing work required.	Response: Thank you for this comment. Community group members will not receive a salary as they intend to function as community volunteers to support local ownership and sustainability of the intervention. We have clarified this in the manuscript.<br><br>Manuscript changes: Page 8, under the heading "Community Education and Awareness".

24	Reviewer 1	CONSORT checklist	Review the CONSORT checklist: some items marked as not applicable are actually addressed in the protocol, for example item 3 on the protocol/registration.	Response: Thank you for this comment. We carefully reviewed the CONSORT checklist and agree that some items had been incorrectly marked as "not applicable." We have revised the checklist accordingly and updated the relevant items with the corresponding page and line numbers where information is reported in the manuscript, including details regarding protocol and trial registration.<br><br>Manuscript changes: None

VERSION 2 - REVIEW

Reviewer 1

Name Herrmann, Alina

Affiliation UniversitatsKlinikum Heidelberg, Institute for Public Health

Date 19-Jun-2026

COI

All comments have been adressed adequately.
